# Two New Synonyms of *Paraleuctra orientalis* (Chu, 1928) (Plecoptera: Leuctridae) Based on Morphological and Molecular Data, with Notes on *Paraleuctra cervicornis* Du and Qian, 2012

**DOI:** 10.3390/insects13050468

**Published:** 2022-05-18

**Authors:** Yu-Ben Yang, Yu-Zhou Du

**Affiliations:** 1 Institute of Applied Entomology, School of Horticulture and Plant Protection, Yangzhou University, Yangzhou 225009, China; yubenyang1998@163.com; 2 Joint International Research Laboratory of Agriculture and Agri-Product Safety, Ministry of Education, Yangzhou University, Yangzhou 225009, China

**Keywords:** Plecoptera, Leuctridae, *Paraleuctra*, synonym, China

## Abstract

**Simple Summary:**

*Paraleuctra* Hanson, 1941, is a widespread genus distributed in the east and west Nearctic and in much of the Palaearctic, with 27 valid species currently known. In China, although six *Paraleuctra* species have been reported, the differences among these species have not been fully explained. After checking a large number of specimens of this genus from Zhejiang Province, two junior synonyms are proposed for the species *Paraleuctra orientalis* (Chu, 1928).

**Abstract:**

We recently examined specimens of the genus *Paraleuctra* Hanson, 1941, from Zhejiang Province and Sichuan Province, China, and two new synonyms are established based on morphological and molecular data. *Paraleuctra sinica* Yang and Yang, 1995, and *Paraleuctra tianmushana* Li and Yang, 2010, are synonymized with *Paraleuctra orientalis* (Chu, 1928). Additionally, we provide new images of *Paraleuctra cervicornis* Du and Qian, 2012, to facilitate identification of this species.

## 1. Introduction

*Paraleuctra* Hanson, 1941, is a widespread genus mainly distributed in the east and west Nearctic and in much of the Palaearctic, with 27 valid species currently known [1,2,3,4,5,6,7,8,9,10,11,12,13,14,15]. The stoneflies of this genus are common inhabitants of stream environments. The nymphs mainly live in cold streams, springs, rock fissure seepage and other water environments with high dissolved oxygen, feeding on mosquito larvae, plant fragments and algae. Their habitat adaptation range is narrow [16]. After emergence into adults, they inhabit the herbs, grasses or shrubs on the bank due to their weak flying ability. Adults live in groups, which is conducive to mating. After mating, the female flies back to the water to lay eggs. Adults can feed on algae, moss, lichens and leaves, allowing them to survive for days to weeks [17,18].

In China, at present, this genus remains poorly known with only six species reported: *P. orientalis* (Chu, 1928), *P. sinica* Yang and Yang, 1995, *P. tianmushana* Li and Yang, 2010, *P. cervicornis* Du and Qian, 2012, *P. qilianshana* Li and Yang, 2013, and *P. cuihuashana* Chen, 2019. The holotype of *P. orientalis* was destroyed and the numbers of the paratypes of *P. sinica* and *P. tianmushana* are small. The differences among these three species are not obvious or unique. The problem of how to identify these three species remains. Therefore, we went to the type locality (province) of these three species for collection to solve this problem.

The mitochondrial cytochrome C oxidase subunit 1 gene (mtCOI) and 18S rDNA of Plecoptera are stable; their gene arrangement is conserved, with few rearrangements, so they have been widely used in evolution, phylogeny, genetics, species identification [8,12,19]. After checking a large number of the specimens of *Paraleuctra* from Zhejiang Province, we established *P. sinica* and *P. tianmushana* as junior synonyms of *P. orientalis* based on the close agreement of morphological characters, COI gene sequence divergence, 18S rDNA sequence divergence and phylogenetic trees of the two genes. Additionally, we provide new images of *P. cervicornis* Du and Qian, 2012, to facilitate identification of this species.

## 2. Material and Methods

### 2.1. Specimen Preparation

Most of the adults of *Paraleuctra* live in the bushes or small trees near the clean and cold streams. Due to their weak flight ability, we used an insect catching net to shake the twigs or the branches, and the adults fell into the net. The adults of *Paraleuctra* have phototaxis, so they can also be collected by a light trapping method. Specimens were preserved in 75% ethanol. Male terminalia specimens were cleared in 10% NaOH. All specimens used in this study are deposited in the Insect Collection of Yangzhou University (ICYZU), Jiangsu Province, China.

### 2.2. Observation and Description

**Figure 1:**
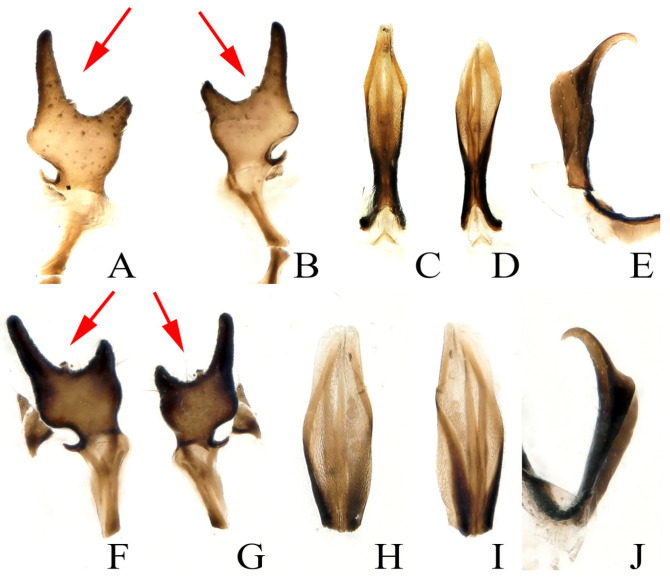
(**A**–**E**) *Paraleuctra tianmushana* Li and Yang, 2010 **syn. nov.**: (**A**,**B**) right cercus of male, lateral view; (**C**) subanal probe of male, dorsal view; (**D**) subanal probe of male, ventral view; (**E**) epiproct of male, lateral view. (**F**–**J**) *Paraleuctra orientalis* (Chu, 1928): (**F**,**G**) right cercus of male, lateral view; (**H**) subanal probe of male, dorsal view; (**I**) subanal probe of male, ventral view; (**J**) epiproct of male, lateral view. (The two specimens were collected from Zhejiang Province, Tianmushan. The arrow points to a small spine on the cerci.)

**Figure 2:**
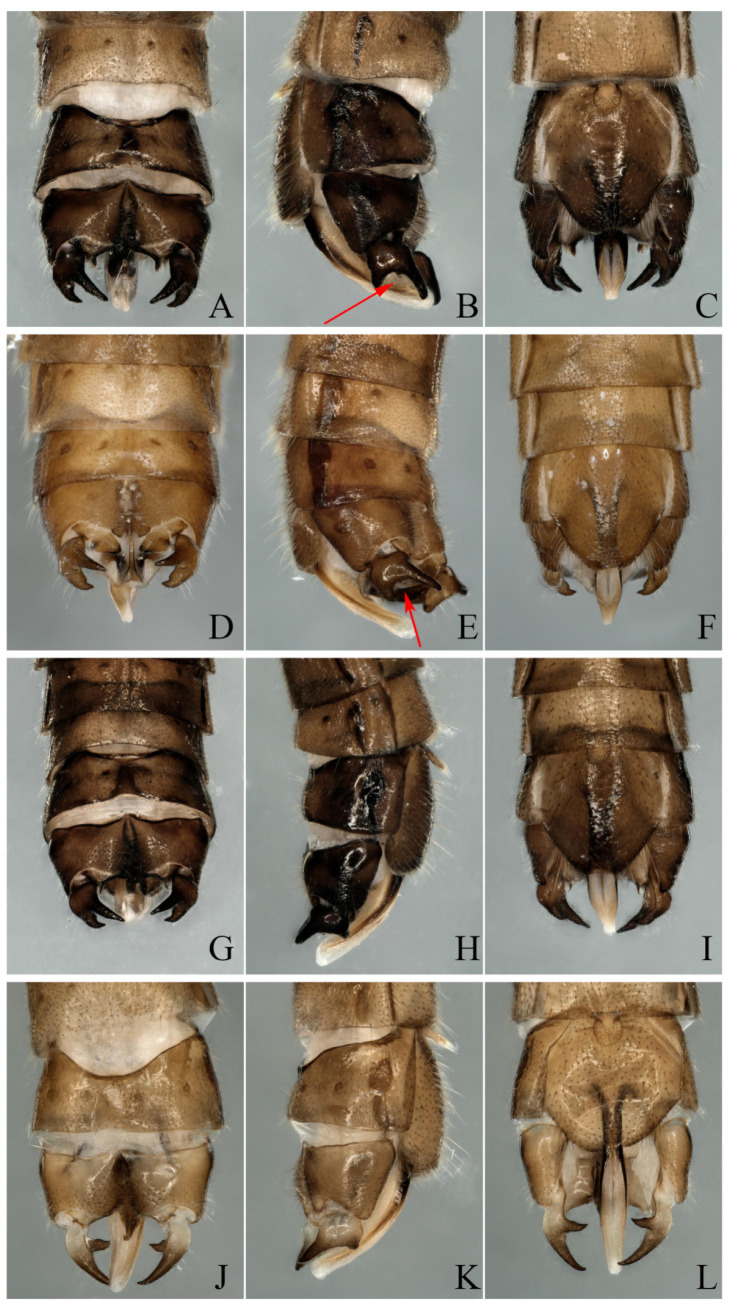
(**A**–**C**) *Paraleuctra orientalis* (Chu, 1928): (**A**) Male terminalia, dorsal view; (**B**) Male terminalia, lateral view; (**C**) Male terminalia, ventral view; (**D**–**F**) *Paraleuctra tianmushana* Li and Yang, 2010 **syn. nov.**: (**D**) Male terminalia, dorsal view; (**E**) Male terminalia, lateral view; (**F**) Male terminalia, ventral view; (**G**–**I**: type B, **J**–**L**: type A) *Paraleuctra sinica* Yang and Yang, 1995 **syn. nov.**: (**G**,**J**) Male terminalia, dorsal view; (**H**,**K**) Male terminalia, lateral view; (**I**,**L**) Male terminalia, ventral view. (The four specimens were collected from Zhejiang Province, Tianmushan. The arrow points to a small spine on the cerci.)

**Figure 3:**
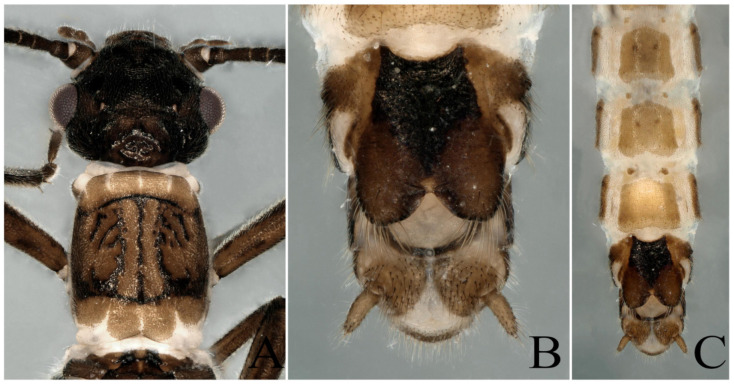
*Paraleuctra orientalis* (Chu, 1928): (**A**) female head and pronotum, dorsal view; (**B**) female terminalia, ventral view; (**C**) female terminalia, ventral view. (This specimen was collected from Zhejiang Province, Tianmushan.)

Morphological details were examined with a Leica MZAPO microscope. Color illustrations were taken with a KEYENCE VHX-5000. The specimens were identified as *Paraleuctra* species by the following features: The *Paraleuctra* species is small, generally no more than 10 mm, and brown or dark brown. At rest, the wings roll into a tube to the abdomen. The subanal probe and epiproct of the male are specialized (Figure 1C–E,H–J). The tergum 10 of the male terminalia is divided into two parts (Figure 2A,D,G,J). Cerci sclerotize and form a dentate process (Figure 1A,B,F,G). The sternum 8 of the female forms an obvious subgenital plate; the cercus is simple with no change (Figure 3B,C). The terminology used for description follows Yang et al. (2015) [20].

### 2.3. DNA Extraction, Amplification and Sequencing

The five specimens used for molecular analyses and morphological identification were collected from Zhejiang Province, Tianmushan. Genomic DNA was extracted using AxyPrep DNA tissue kits (America), following the manufacturer’s instruction.

A 1745 bp fragment covering the entire mitochondrial COI gene was amplified using a pair of specific primers [8] (F: AAACTAATAGCCTTCAAAG, R: TATWTGGAGCTTAAATCCAT) with 50 °C annealing temperature and 35 PCR cycles. PCR products were purified and sequenced by Biozeron Biotech Co. (Shanghai, China) in both directions using the same primers as above. All sequences of the COI gene were aligned using ClustalW and then cut to 658 bp for analyses.

A 491 bp fragment of 18S rDNA was amplified using a pair of primers [19] (18S a0.7: ATTAAAGTTGTTGCGGTT, 18S b2.9: TATCTGATCGCCTTCGAACCTCT). PCR products were purified and sequenced by Biozeron Biotech Co. (Shanghai, China) in both directions using the same primers as above. All sequences of 18S rDNA were aligned using ClustalW. The sequences were deposited in GenBank under accession numbers, as listed in Table 1.

### 2.4. Phylogenetic Analyses

To root the *Paraleuctra* section of the trees, we included as outgroups representatives of *Leuctra*, *Perlomyia* and *Rhopolopsole* available from GenBank (Table 1). We also added other species of *Paraleuctra* from either the Palearctic or Nearctic to add context to our target *Paraleuctra* species. Note that the outgroups and additional Paraleuctra species varied by gene used, necessitating the separation of genes for analyses.

The genetic distance and maximum likelihood (ML) analysis were computed using MEGA v. 7.0. The parameters of ML analysis are as follows—test of phylogeny: bootstrap method with 1000 bootstrap replications; substitution type: nucleotide; model/method: Kimura 2-parameter model; rates among sites: uniform rates; gaps/missing data treatment: complete deletion; ML heuristic method: nearest-neighbor-interchange (NNI); initial tree for ML: make initial tree automatically (Default-NJ/BioNJ); branch swap filter: none.

MrBayes v. 3.1.2 was utilized to generate the topology for the Bayesian inference (BI) analysis, the working procedure is as follows:

execute XXX.nex

outgroup XX, XX

lset nst = 6 rate = invgamma

showmodel

mcmc ngen = 100000 samplefreq = 100

If the value is greater than 0.01, continue; if it is less than 0.01, enter: sump burnin = 250 (total running generation/sampling frequency then divided by 4) sumt burnin = 250

## 3. Results

### 3.1. Paraleuctra orientalis (Chu, 1928)

*Leuctra orientalis*: Chu, 1928: 87 [2].

*Rhopalopsole orientalis*: Illies, 1966: 118 [21].

*Paraleuctra orientalis*: Zwick, 1973: 410 [22]; Du and Sivec, 2005: 40 [23]; Li et al. 2010: 47 [5]; Du and Qian, 2012: 1062 [6]; Qian and Du, 2012: 3 [24]; Qian et al. 2014: 606 [25]; Yang et al. 2015: 84 [20]; Chen, 2019: 237 [8].

*Paraleuctra sinica***syn. nov.**: Yang and Yang, 1995: 24 [4]; Li et al. 2010: 47 [5]; Du and Qian, 2012: 1062 [6].

*Paraleuctra tianmushana***syn. nov.**: Li et al. 2010: 47 [5]; Du and Qian, 2012: 1062 [6]; Li et al. 2016: 94 [26].

**Type locality:** China, Zhejiang Province (Lin–an).

**Material examined:** 2 males, China, Zhejiang Province, Longwangshan, 1999-Ⅵ-4, leg. DU Yu-Zhou (ICYZU); 21 males, 51 females, China, Zhejiang Province, Tianmushan, 2006-Ⅲ-19, leg. WANG Zhi-Jie (ICYZU); 53 males, 63 females, China, Zhejiang Province, Tianmushan, 378.36 m, N: 30.2118, E: 119.283, 2021-Ⅲ-25, leg. HUO Qing-Bo, ZHAO Meng-Yuan and XIANG Ya-Nan (ICYZU).

**Distribution:** China (Henan, Shaanxi, Anhui, Hubei, Sichuan, Yunan, Zhejiang, Fujian); Russia.

**Figure 4:**
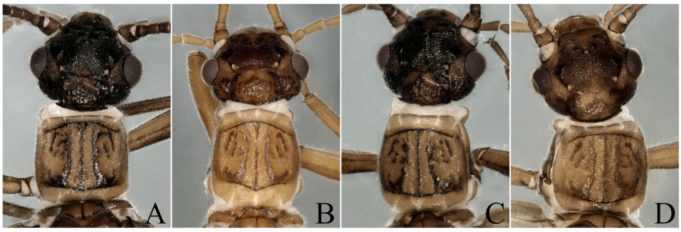
(**A**) *Paraleuctra orientalis* (Chu, 1928): male head and pronotum, dorsal view; (**B**) *Paraleuctra tianmushana* Li and Yang, 2010 **syn. nov.**: male head and pronotum, dorsal view; (**C**: type B, **D**: type A) *Paraleuctra sinica* Yang and Yang, 1995 **syn. nov.**: male head and pronotum, dorsal view. (The four specimens were collected from Zhejiang Province, Tianmushan).

**Remarks:** At present, only six species of the genus *Paraleuctra* Hanson, 1941, are reported in China. *Pareleuctra orientalis* is widespread in China; the adults are dark brown with uniform body color, cerci somewhat sclerotized, strongly forked into two sharp prongs; the upper prong is longer than the lower prong; a spine is present near base of upper prong, projecting backwards (Figure 1F,G, Figure 2A–C and Figure 4A). Yang et al. (2015) said that *P. orientalis* can be distinguished from *P. tianmushan* as the body color of *P. tianmushan* is yellowish brown. The difference between *P. orientalis* and *P. sinica* should be that the male cerci of *P. sinica* lacks a small bulge on the dorsal arm, tergum 9 with sclerotized median strips; the female subgenital plate lobes are slightly sclerotized, with the posterior margin more roundly expanded [4,5,20].

**Results and Discussion:** The holotype of *P. orientalis* was destroyed and the numbers of the paratypes of *P. sinica* and *P. tianmushana* are small. The differences among these three species are not obvious or unique. The problem of how to identify these three species remains. In order to solve this problem, we went to the type locality (province) to collect these three species many times. After checking a large number of specimens of this genus from Zhejiang Province, we have evidence that *P. sinica* and *P. tianmushana* are junior synonyms of *P. orientalis*:

After examining the specimens from Zhejiang Province, according to the original descriptions [4,5,20], we identified the specimen shown in Figure 2A–C and Figure 4A as *P. orientalis*; Figure 2D–F and Figure 4B as *P. tianmushana*; Figure 2G–I and Figure 4C (type B: the body color of this specimen is dark brown); Figure 2J–L and Figure 4D (type A: the body color of this specimen is yellowish brown) as *P. sinica*; although the types of *P. sinica* have not been examined, we can identified these two specimens as *P. sinica* by the specimens lacking a bulge on the cerci (Figure 2J–L) or having an almost invisible spine on the dorsal arm (Figure 2G–I).

**Figure 5:**
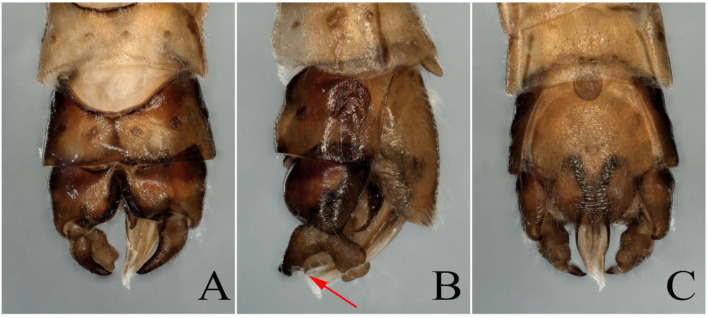
*Paraleuctra cervicornis* Du and Qian, 2012: (**A**) male terminalia, dorsal view; (**B**) male terminalia, lateral view; (**C**) male terminalia, ventral view. The specimen was collected from Sichuan Province (Mt Niba). The arrow points to a small spine on the cerci.

According to the identification results, we further found that the *Paraleuctra* specimens from the same site (Tianmushan) had two types of cerci: A spine present near the base of the upper prong (Figure 1F,G and Figure 2B,E), another type without a spine (Figure 2K) or with an almost invisible spine (Figure 2H); two types of color patterns of the body (Figure 1, Figure 2, Figure 3 and Figure 4); the same patterns of epiproct and subanal probe (Figure 1C–E,H–J and Figure 2); all the female specimens had only one pattern of subgenital plates (Figure 3B,C). When we dissected the cercus of the specimens without a spine from all views (Figure 2J–L), the cercus also had an almost invisible spine near the base of the upper prong (Figure 1A,B). It turns out that different color patterns, with or without a spinule can be found on the same species, and this result could overturn the identification basis [20] used to distinguish these three species. Therefore, we have come to the conclusion that the difference in color patterns or spinules consists merely of individual variation and, to some degree, the age of the specimens. Additionally, we think without examining a large number of specimens of the species from this genus, the differences among all species cannot be fully explained, unless a species has unique character, such as the shape of the cerci of *P. cervicornis*, which is like an antler (Figure 5B), and differs from all other *Paraleuctra* species.

**Figure 6:**
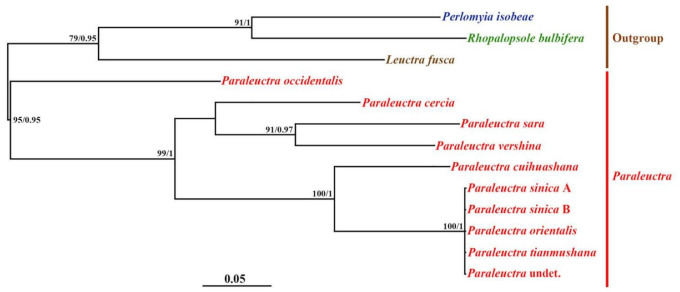
Phylogenetic trees of COI gene. Phylogenetic relationships among 13 stoneflies based on maximum likelihood (ML) analysis and Bayesian inference (BI) tree (numbers at the nodes are ML bootstrap values (**left**) and Bayesian posterior probabilities (**right**); only nodal support values of >70% are depicted on the tree). The scale bar represents the rate of base substitutions.

**Figure 7:**
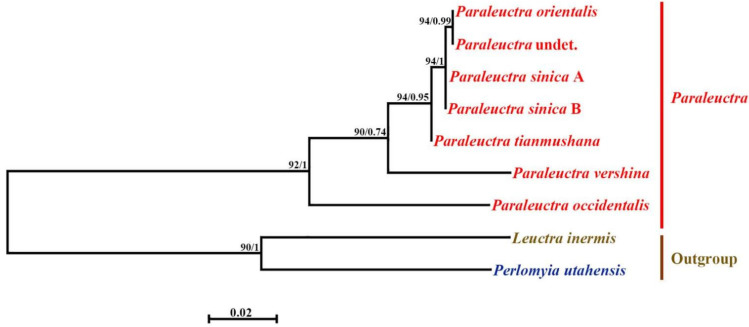
Phylogenetic trees of 18S rDNA. Phylogenetic relationships among 9 stoneflies based on maximum likelihood (ML) analysis and Bayesian inference (BI) tree (numbers at the nodes are ML bootstrap values (**left**) and Bayesian posterior probabilities (**right**), only nodal support values of >70% are depicted on the tree.) The scale bar represent the rate of base substitutions.

The genetic distance data and phylogenetic analyses using gene sequence data both provide ample evidence for the new junior synonym status of *P. sinica* and *P. tianmushana* (Table 1, Table 2, Table 3 and Table 4, Figure 6 and Figure 7). The genetic distance of the COI gene among the specimens of these three species is 0% and the value is lower than the commonly used 3% for species delimitation with DNA barcoding [27,28]. The genetic distance of 18S rDNA among the specimens of these three species ranges 0–1% also supports the synonymy of *P. sinica* and *P. tianmushana* with *P. orientalis* [29]. We also sequence the COI gene and 18S rDNA for one female collected from the same sites, and the genetic distances among the five specimens are also below the commonly used values.

In the maximum likelihood (ML) and Bayesian inference (BI) trees (Figure 6 and Figure 7), *P. orientalis*, *P. tianmushan*, *P. sinica* type A, type B (A, B represent different color patterns) and *P.* sp. (a female one) are grouped in the same clade, separated from the clade of other *Paraleuctra* species. The topology of the ML tree and BI tree are highly supported, with high support values on most nodes. Additionally, in terms of specimen collection location, it supports that specimens collected from the same place belong to the same species. At the genus level, the monophyly of the genus is supported by all phylogenetic trees (Figure 6 and Figure 7). Among them, the phylogenetic relationship between *Paraleuctra* and *Leuctra* is the closest, and the genetic distance between the 18S rDNA of *Paraleuctra* and *Perlomyia* is the largest (Table 3), indicating that the 18S rDNA may have obvious sequence differences between *Paraleuctra* and *Perlomyia*. The genetic distance of the COI gene among the specimens collected from China is below 12%; in contrast to the specimens of *Paraleuctra* not collected from China, this value is over 15% (Table 2). In addition, in the phylogenetic trees, the clades of them separated from the clades of the specimens of *Paraleuctra* not collected from China (Figure 6 and Figure 7). The genetic distances of 18S rDNA among the *Paraleuctra* species are below 10% (Table 3); it seems that 18S rDNA is more stable than COI gene of species in the genus *Paraleuctra*. Due to the limited samples, the phylogenetic status of *Rhopalopsole*, *Leuctra* and *Perlomyia* still require further study by supplementary sampling.

Molecular data used in this study have proved their efficiency in identifying the morphologically similar species, such as those in the genus *Paraleuctra*. Combined morphological and molecular evidence (Table 4), we propose that *P. sinica* and *P. tianmushana* are junior synonyms of *P. orientalis*.

### 3.2. Paraleuctra cervicornis Du and Qian, 2012

*Paraleuctra cervicornis*: Du and Qian, 2012: 1059 [6].

**Type locality:** China, Sichuan Province (Mt Niba).

**Material examined:** Holotype (male): Mt Niba (N: 29.6644, E: 102.6039, 2250 m), Yingjing county, Sichuan Province, 1996-Ⅵ-17, Leg. DU Yu-Zhou; Paratypes: 1 male and 4 females same data as holotype; 1 male and 2 females, Mt Erlang, N: 29.8486, E: 102.3045, 2380 m), Tianquan County, Sichuan Province, 1996-Ⅵ-7, Leg. DU Yu-Zhou.

**Distribution:** Russia; China (Henan, Shaanxi, Anhui, Hubei, Sichuan, Yunan, Zhejiang, Fujian).

**Figure 8:**
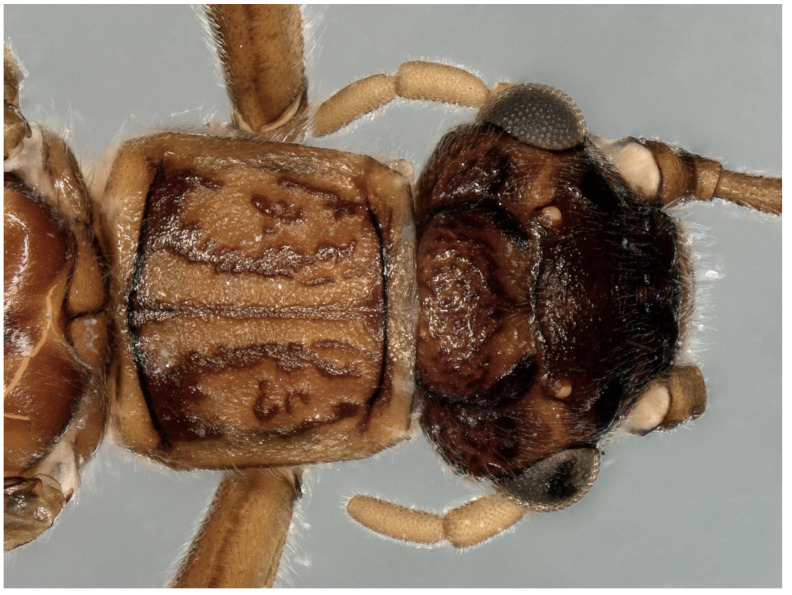
*Paraleuctra cervicornis* Du and Qian, 2012: male head and pronotum, dorsal view. The specimen was collected from Sichuan Province (Mt Niba).

**Figure 9:**
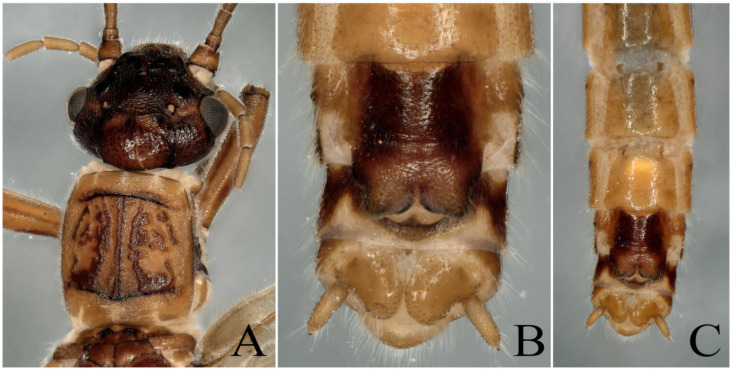
*Paraleuctra cervicornis* Du and Qian, 2012: (**A**) female head and pronotum, dorsal view; (**B**) female terminalia, ventral view; (**C**) female terminalia, ventral view. The specimen was collected from Sichuan Province (Mt Niba).

**Supplementary Description**: This species had been well described by Du and Qian [6]. In this study, we provide the color figures of this species to facilitate identification. The heads of the males and females of this species are much flatter than the pronotum (Figure 8 and Figure 9A). Cerci strongly forked into two prongs, the upper prong sharp, pointed and bearing a small, ventrally directed mesal spine, whereas the lower prong is rounded or somewhat truncate with an emarginated apex (Figure 5B).

**Remarks:** The shape of cerci in the male is unique among all Asian *Paraleuctra* species. It seems that all the males of *Paraleuctra* species in China have a spine on the cercus, but it is obvious in some specimens (Figure 1F,G, Figure 2B,E and Figure 5B) and not in some (Figure 1A,B and Figure 2H,K). The subgenital plate of the female of this species can also be distinguished from all other species from China in this genus by the lobes being subrectangular.

## Figures and Tables

**Table 1:** List of taxa included in this analysis.

Genus	Species	GenBank Accession No.	
		COI Gene	18S rDNA
*Paraleuctra*	*orientalis*	OM836700	ON130265
*Paraleuctra*	*sinica* A	OM836702	ON130263
*Paraleuctra*	*sinica* B	OM203120	ON130264
*Paraleuctra*	*tianmushana*	OM836701	ON130266
*Paraleuctra*	sp. F	OM836704	ON130267
*Paraleuctra*	*cuihuashana*	MK995183	
*Paraleuctra*	*cercia*	MK492251	
*Paraleuctra*	*vershina*	MH840222	AY521878
*Paraleuctra*	*sara*	HQ938243	
*Paraleuctra*	*occidentalis*	MG380092	EF622723
*Perlomyia*	*isobeae*	MK492252	
*Perlomyia*	*utahensis*		EF622724
*Rhopalopsole*	*bulbifera*	MK111419	
*Leuctra*	*fusca*	MK568475	
*Leuctra*	*inermis*		EF622721

Note: F represents an undetermined female; A indicates the body color of this specimen is yellowish brown; B indicates the body color of this specimen is dark brown (the same as below).

**Table 2:** Genetic distance of the COI gene (nucleotides) among the species used in this study.

	Genetic Distance (%)											
Name	*P. orientalis*	*P. sinica* A	*P. sinica* B	*P. tianmushana*	*P. undet.*	*P. cuihuashana*	*P. cercia*	*P. vershina*	*P. sara*	*P. occidentalis*	*P. isobeae*	*R. bulbifera*
*P. orientalis*												
*P. sinica A*	0.0											
*P. sinica B*	0.0	0.0										
*P. tianmushana*	0.0	0.0	0.0									
*P. undet.*	0.0	0.0	0.0	0.0								
*P. cuihuashana*	11.7	11.7	11.7	11.7	11.7							
*P. cercia*	18.3	18.3	18.3	18.3	18.3	15.3						
*P. vershina*	17.4	17.4	17.4	17.4	17.4	17.1	14.8					
*P. sara*	19.5	19.5	19.5	19.5	19.5	19.4	14.7	13.0				
*P. occidentalis*	20.4	20.4	20.4	20.4	20.4	20.7	18.8	19.3	19.8			
*P. isobeae*	24.3	24.3	24.3	24.3	24.3	22.8	19.5	21.7	21.1	19.0		
*R. bulbifera*	24.9	24.9	24.9	24.9	24.9	23.2	20.0	22.7	23.3	20.5	15.4	
*L. fusca*	23.0	23.0	23.0	23.0	23.0	22.6	22.6	24.4	23.6	19.6	19.9	20.1

**Table 3:** Genetic distance of the 18S rDNA (nucleotides) among the species used in this study.

	Genetic Distance (%)							
Name	*P. orientalis*	*P. sinica* A	*P. sinica* B	*P. tianmushana*	*P.* *undet.*	*P. vershina*	*P. occidentalis*	*P. utahensis*
*P. orientalis*								
*P. sinica* A	0.2							
*P. sinica* B	0.2	0.0						
*P. tianmushana*	0.6	0.4	0.4					
*P. undet.*	0.0	0.2	0.2	0.6				
*P. vershina*	5.6	5.4	5.4	4.9	5.6			
*P. occidentalis*	9.5	9.3	9.3	8.8	9.5	9.0		
*P. utahensis*	30.3	30.0	30.0	29.3	30.3	30.9	29.3	
*L. inermis*	28.0	27.7	27.7	27.0	28.0	24.8	26.9	14.8

**Table 4:** Methods used to prove *P. sinica* and *P. tianmushana* are junior synonyms of *P. orientalis* in this study.

Species Name	Methods				
	Morphology	Genetic Distance of the COI Gene	Genetic Distance of the 18S rDNA	Phylogenetic Trees of COI Gene	Phylogenetic Trees of 18S rDNA
*P. orientalis*	Share similar features such as head, pronotum, male terminalia and female terminalia.	Genetic distance of the COI gene among the three species is below 2%.	Genetic distance of the 18S rDNA is below 2%.	The three species are grouped in the same clade with high support values.	The three species are grouped in the same clade with high support values.
*P. sinica A*					
*P. sinica B*					
*P. tianmushana*					
*P. undet.*					

## Data Availability

All data is available in this paper.

## References

[1] DeWalt R.E., Maehr M.D., Neu-Becker U., Stueber G. Plecoptera Species File Online. http://Plecoptera.SpeciesFile.org.

[2] Chu Y.T. (1928). Description of a new species of *Leuctra* and notes on *Nemoura sinensis* from Hangchow. China J..

[3] Hanson J.F. (1941). Studies on the Plecoptera of North America. Bullet. Brooklyn Entomol. Soc..

[4] Yang D., Yang C.K., Zhu T. (1995). Plecoptera: Leuctridae. Insects and Macrofungi of Gutianshan, Zhejiang.

[5] Li W.H., Wang Y.B., Yang D. (2010). Synopsis of the genus *Paraleuctra* (Plecoptera: Leuctridae) from China. Zootaxa.

[6] Du Y.Z., Qian Y.H. (2012). *Paraleuctra cervicornis* (Plecoptera: Leuctridae), a new stonefly from China. J. Nat. Hist..

[7] Kong F.B., Yang D., Li W.H. (2013). Discovery of the genus *Paraleuctra* (Plecoptera: Leuctridae) from Palearctic China, with description of *P. qilianshana* sp. nov.. Zootaxa.

[8] Chen Z.T. (2019). The first formal report of brachypterous stonefly of Leuctridae (Plecoptera) from China. Zootaxa.

[9] Zhiltzova L.A. (1974). Rare genera of the family Leuctridae (Insecta: Plecoptera) in the fauna of the USSR. Zool. Zhurnal.

[10] Harper P.P. (1977). Capniidae, Leuctridae, and Perlidae (Plecoptera) from Nepal. Orient. Insects.

[11] Shimizu T. (2000). *Paraleuctra* (Insecta: Plecoptera: Leuctridae) from Japan, with taxonomic notes on the Japanese Leuctridae. Species Divers..

[12] Stark B.P., Juliana W.K. (2001). Systematics of Nearctic *Paraleuctra* with description of a new genus (Plecoptera: Leuctridae). Tijdschr. Entomol..

[13] Cherchesova S.K., Zhiltzova L.A. (2003). The stonefly fauna (Plecoptera) of North Ossetia and its zoogeographical characteristics. Entomol. Rev..

[14] Baumann R.W., Stark B.P. (2009). *Paraleuctra alta* (Plecoptera: Leuctridae), a new stonefly from Alberta, Canada. Illiesia.

[15] Murányi D., Hwang J.M. (2017). Four new species and further contributions to the Leuctridae (Plecoptera) of the Korean Peninsula. Zootaxa.

[16] William D.D., Feltmate B.W. (1992). Chapter V–Order Plecoptera. Aquatic Insects.

[17] Zwick P. (1996). Variable egg development of *Dinocras* spp. (Plecoptera, Perlidae) and the stonefly seed banktheory. Freshw. Biol..

[18] Snellen R.K., Stewart K.W. (1979). The life cycle and drumming behavior of *Zealeuctra claasseni* (Frison) and *Zealeuctra hitei* (Ricker and Ross) (Plecoptera: Leuctridae) in Texas, USA. Aquatic Insects.

[19] Terry M.D., Whiting M.F. (2005). Mantophasmatodea and phylogeny of the lower neopterous insects. Cladistics.

[20] Yang D., Li W.H., Zhu F. (2015). Fauna Sinica, Insecta. Plecoptera: Nemouroidea.

[21] Illies J. (1966). Katalog der rezenten Plecoptera. Das Tierreich.

[22] Zwick P. (1973). Insecta: Plecoptera, Phylogenetisches System und Katalog. Das Tierreich.

[23] Du Y.Z., Sivec I., Yang X.K. (2005). Insect Fauna of Middle-west Qinling Range and South Montains of Gansu Province.

[24] Qian Y.H., Du Y.Z. (2012). A new stonefly species, *Rhopalopsole tricuspis* (Leuctridae: Plecoptera), and three new records of stoneflies from the Qinling Mountains of Shaanxi, China. J. Insect Sci..

[25] Qian Y.H., Li H.L., Du Y.Z. (2014). A Study of Leuctridae (Insecta: Plecoptera) from Shennongjia, Hubei Province, China. Fla. Entomol..

[26] Li W.H., Li K.F., Wang R.F., Yang D. (2016). The first description of the larvae of the Chinese species *Paraleuctra tianmushana* Li & Yang (Plecoptera: Leuctridae). Zootaxa.

[27] Hebert P.D., Ratnasingham S., de Waard J.R. (2003). Barcoding animal life: Cytochrome c oxidase subunit 1 divergences among closely related species. Proc. R. Soc. Lond. Ser. B Biol. Sci..

[28] Zhou X., Adamowicz S.J., Jacobus L.M., DeWalt R.E., Hebert P.D. (2009). Towards a comprehensive barcode library for Arctic life-Ephemeroptera, Plecoptera, and Trichoptera of Churchill, Manitoba, Canada. Front. Zool..

[29] Baur A., Buschinger A., Zimmermann F.K. (1993). Molecular cloning and sequencing of 18S rDNA gene fragments from six different ant species. Insectes Sociaux.

